# A Rare Case of Type B Neonatal Pyruvate Carboxylase Enzyme Deficiency Presenting With Refractory Lactic Acidosis in the Early Neonatal Period

**DOI:** 10.7759/cureus.29903

**Published:** 2022-10-04

**Authors:** Saima Sharif, Pradeep Kumar Velumula, Praveen kumar Boddu, Deniz Altinok, Nithi Fernandes

**Affiliations:** 1 Pediatrics, Neonatal-Perinatal Medicine, Children's Hospital of Michigan/Central Michigan University College of Medicine, Detroit, USA; 2 Pediatrics, Neonatal-Perinatal Medicine, MercyOne Waterloo Medical Center, Waterloo, USA; 3 Radiology, Children's Hospital of Michigan, Detroit, USA

**Keywords:** lactate peak, magnetic resonance spectroscopy (mrs), pyruvate carboxylase enzyme deficiency, pyruvate carboxylase, acidosis lactic, term neonate

## Abstract

Pyruvate carboxylase (PC) enzyme deficiency is a rare genetic disorder inherited in an autosomal recessive (AR) manner. PC, a mitochondrial enzyme, converts pyruvate to oxaloacetate (OAA), which enters the tricarboxylic acid (TCA) cycle. Based on the tissue type, intermediate metabolites of the TCA cycle play a vital role in gluconeogenesis, lipogenesis, synthesis of nicotinamide adenine dinucleotide phosphate (NADPH), and neurotransmitter glutamate in the astrocytes. The severity of clinical presentation depends on the type of PC deficiency and on the residual enzyme activity. We present a term female infant admitted with refractory lactic acidosis that developed soon after birth. On biochemical evaluation, serum ammonia was 125 µmol/L; plasma amino acid analysis showed elevated citrulline, lysine, proline, decreased glutamine, and aspartic acid; urine organic acid analysis showed markedly increased lactic acid, and moderately elevated 3-hydroxy-butyric and acetoacetic acid. MRI brain demonstrated abnormal diffuse white matter edema, loculated and septate large cysts along the caudothalamic notch as well as lateral aspect of the frontal horn bilaterally. Magnetic resonance (MR) spectroscopy showed large amounts of lactate peak. Molecular genetic analysis showed two pathogenic variants in the PC* *gene confirming the diagnosis of PC enzyme deficiency. The infant was discharged home on palliative and hospice care, and she died on the 22^nd^ day after birth.

## Introduction

Pyruvate carboxylase (PC) enzyme deficiency is inherited as an autosomal recessive disorder with an estimated incidence of one in 250,000 births [1]. PC enzyme is a biotin containing mitochondrial enzyme that catalyzes the carboxylation of pyruvate to oxaloacetate (OAA). OAA enters tricarboxylic acid (TCA) cycle, and the intermediate metabolites are involved in gluconeogenesis, lipogenesis, synthesis of nicotinamide adenine dinucleotide phosphate (NADPH), and neurotransmitter glutamate in the astrocytes. PC deficiency is caused by mutations in the PC gene. Depending on the residual activity of PC enzyme, three types of clinical presentations have been described with variation in severity of clinical, biochemical manifestations and prognosis. We report a rare case of neonatal or Type B PC enzyme deficiency presenting soon after birth with severe lactic acidosis in a term female infant.

## Case presentation

A term female neonate was transferred to our neonatal intensive care unit (NICU) for the management of respiratory distress and metabolic acidosis which developed soon after birth. The infant’s mother is a 37-year-old Caucasian and had regular prenatal care. Other than well-controlled gestational diabetes and hypothyroidism, her pregnancy was uneventful. The non-invasive prenatal screening test was reported as low risk and fetal ultrasounds were all reported normal. Prenatal blood work was unremarkable. She had a non-consanguineous relationship with the biological father and no history of spontaneous abortions or known genetic disorders in both extended families. The neonate was born by scheduled Cesarean section at 39 weeks and four days post-menstrual age. She received routine newborn care after delivery and her Appearance, Pulse, Grimace, Activity, and Respiration (APGAR) scores were eight and nine at one and five minutes after birth, respectively. Her birth weight was 2,795 grams (15th percentile), length was 51.4 cm (89th percentile), and head circumference was 35.6 cm (93rd percentile). A few hours after birth, she was admitted to the neonatal intensive care unit (NICU) for respiratory distress. On examination, she was tachypneic with labored breathing, with bilateral clear breath sounds, no cardiac murmur, good skin perfusion, and no dysmorphic features. She was started on a heated high-flow nasal cannula (HHFNC) while a sepsis screen was performed, then started on antibiotics and intravenous fluids. Her chest radiograph showed bilateral streaky opacities suggestive of transient tachypnea of the newborn and initial arterial blood gas noted severe metabolic acidosis with a pH of 7.07, base deficit -18.9, and lactate of 14 mg/dl. An echocardiogram revealed no structural or functional abnormality. Initial laboratory work (Table 1) was mostly within normal limits except for metabolic acidosis with low bicarbonate with initial respiratory compensation. Her serial blood gas results are shown in Table 2.

**Table 1:** Initial laboratory work from day one after birth

Variable	Patient's value	Reference value
Serum Alanine transaminase (ALT)	9 units/L	7-52 units/L
Serum Aspartate transaminase (AST)	49 units/L	11-39 units/L
Blood urea nitrogen	17 mg/dL	7- 25 mg/dL
Creatinine	0.84 mg/dL	0.79- 1.3 mg/dL
WBC	20.4 k/cumm	4.5-25 k/cumm
Neutrophils %	74 %	15-78 %
Absolute neutrophil count (ANC)	13.5 k/cumm	1.58-7.13 k/cumm
Sodium	143 mmol/L	136-145 mmol/L
Potassium	4.3 mmol/L	3.5-5.1 mmol/L
Chloride	111 mmol/L	98-107 mmol/ L
Bicarbonate	7 mmol/L	21-31 mmol/L
Serum Calcium	9.7 mg/dL	8.6-10.3 mg/dL
C reactive protein	< 5 mg/L	< 5mg/L

**Table 2:** Blood gas trend over the hospital course PCO2=partial pressure of carbon dioxide; O2=oxygen; HCO3=bicarbonate

Date	Source of blood sample	pH	PCO2	O2	HCO3	LACTATE
Day 2	Arterial	7.23	17	52	7.4	15.4
Day 3	Arterial	7.42	16	103	13	13.68
Day 5	Capillary	7.44	24	74	15.8	11.45
Day 6	Capillary	7.48	28.7	50	21	8.97
Day 10	Capillary	7.50	31.5	78.9	24	10.28

Urine analysis was within normal limits and was negative for reducing substances. She was transferred to our tertiary care level IV NICU for further management of lactic acidosis and to rule out metabolic disorders.

She continued on HHFNC respiratory support and received acetate in intravenous fluids for ongoing metabolic acidosis. Antibiotics were discontinued as the blood culture was negative. The genetics team was consulted as initial labs showed serum ammonia of 125 µmol/L, but urine organic acid analysis showed markedly increased lactic acid and moderately increased 3-hydroxy-butyric acid and acetoacetic acid; and the plasma amino acid profile noted elevated citrulline, lysine, proline, and decreased glutamine and aspartic acid, which was highly suspicious for PC enzyme deficiency. Along with recommendations and close follow-up by pediatric genetics, she was started on oral sodium citrate, biotin, and triheptanoin. Her head ultrasound showed abnormal intraventricular septations and cystic changes at the caudothalamic grooves with prominent echogenic bilateral choroid plexus and accentuation of the white matter echogenicity with a smooth abnormal sulcation pattern. 

**Figure 1:**
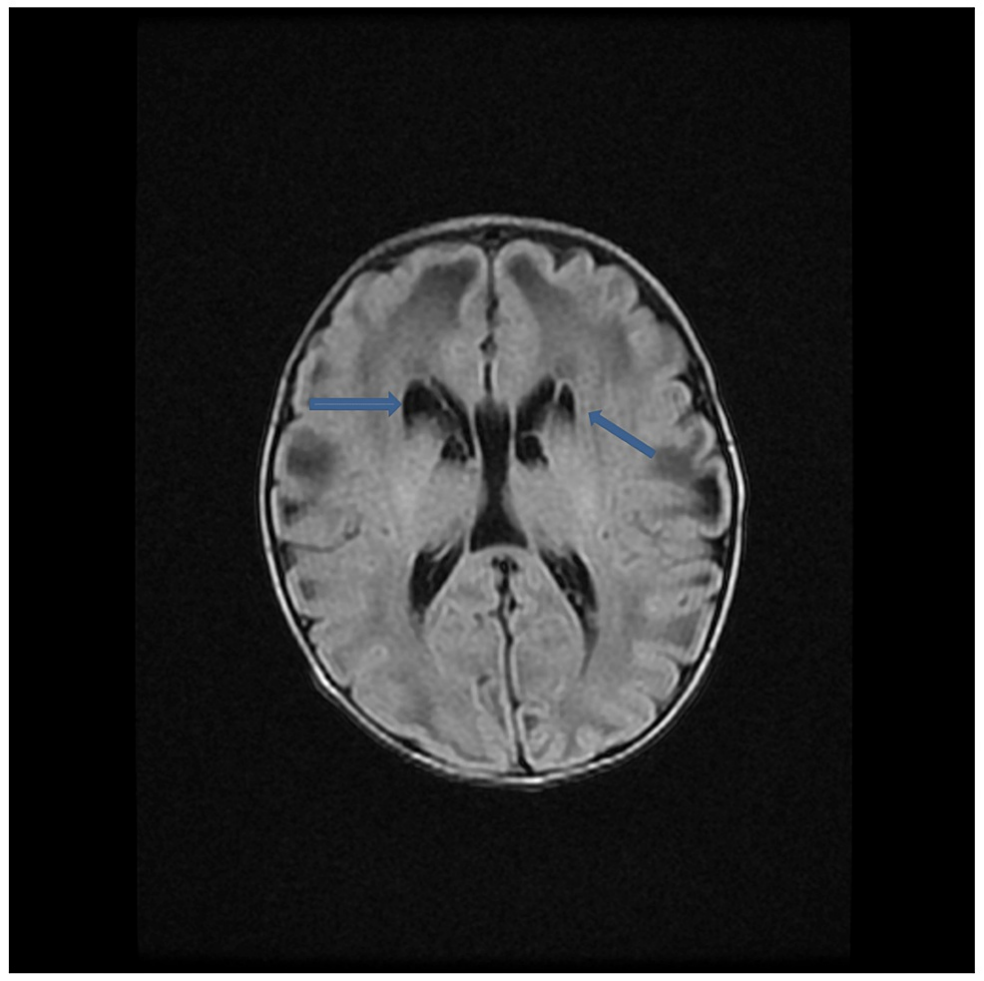
Axial T2-Flair sequence shows multiple subependymal-germinolytic periventricular cysts. Bilateral subcortical parenchymal cysts are also noted in bilateral parietal lobe left more than right. Blue solid arrows point to periventricular cysts

**Figure 2:**
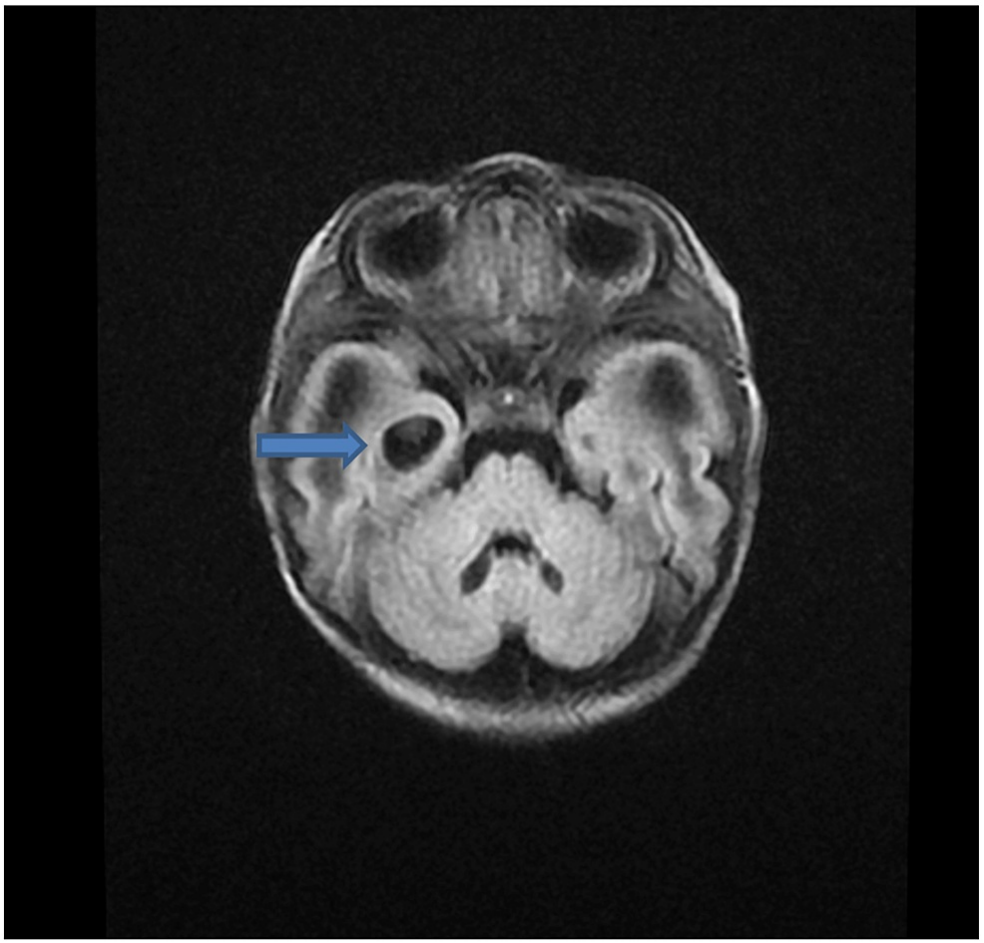
Axial T2-Flair sequence shows subependymal-parenchymal cyst in right temporal pole Blue solid arrow points to subependymal-parenchymal cyst

**Figure 3:**
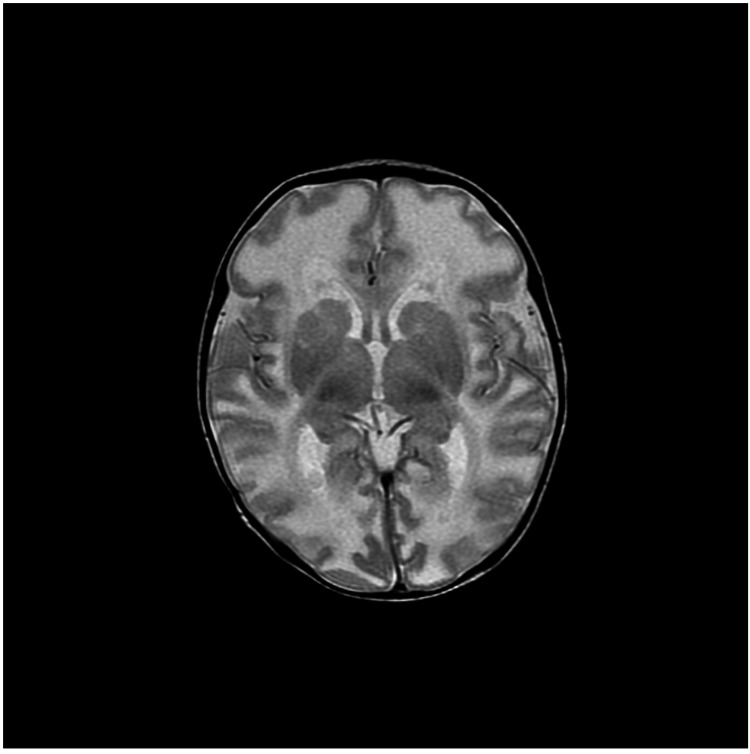
Axial T2 W sequence shows diffuse abnormal signal in hemispheric deep and subcortical white matter

**Figure 4:**
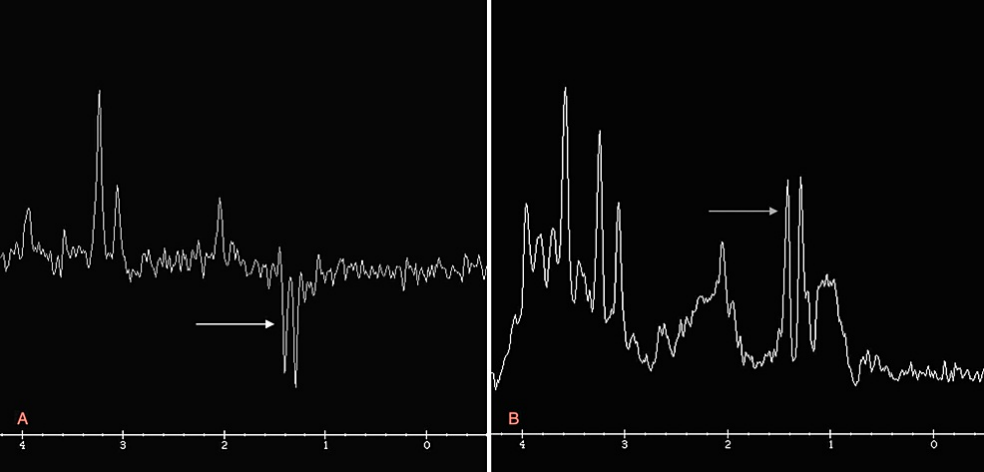
MR spectroscopy shows significant elevation of choline and diminished N-Acetyl aspartate (A) and creatinine peak with large Lactate peak (B) Arrow head in Panel A: N-Acetyl aspartate. Arrow head in Panel B: Lactate

MRI brain demonstrated abnormal diffuse white matter edema involving the frontal temporoparietal occipital deep and subcortical white matter with associated loculated and septate large cysts along the caudothalamic notch as well as lateral aspect of the frontal horn bilaterally (Figure 1). The axial T2-Flair sequence showed a sub-ependymal-parenchymal cyst in the right temporal pole (Figure 2). Axial T2 W sequence showed diffuse abnormal signal in hemispheric deep and subcortical white matter (Figure 3). MR spectroscopy demonstrated diminished amounts of N-Acetyl aspartate (Figure 4A) and large amounts of lactate peak (Figure 4B). On molecular genetic analysis, compound heterozygous pathogenic variations were identified. It showed two pathogenic variants in the PC gene: c.1825+2T>C (splice site, maternally inherited) and c.1556delC (paternally inherited) confirming the diagnosis of PC enzyme deficiency. Over the next few days of the hospital course as her condition deteriorated, following a multidisciplinary meeting, her parents chose palliative and hospice care. She was discharged home after nine days, and she passed peacefully amongst her family at home at 22 days.

## Discussion

We report a rare case of neonatal or type B PC enzyme deficiency presenting soon after birth with severe lactic acidosis in a term female infant. PC enzyme deficiency is inherited as an autosomal recessive disorder with an estimated incidence of one in 250,000 births. [1] The PC enzyme is a biotin-containing mitochondrial enzyme that catalyzes the carboxylation of pyruvate to OAA. OAA enters the Krebs tricarboxylic acid cycle, and based on the tissue type, the intermediate metabolites of the cycle are utilized for various biosynthetic pathways. In the liver, OAA is utilized as a precursor for gluconeogenesis [2,3]; in the adipose tissues, it plays a crucial role in lipogenesis [4]; in the pancreatic tissues, through the pyruvate cycle, is involved in the production of NADPH [5]; and in the astrocytes, neurotransmitter glutamate, is synthesized from alpha-ketoglutarate, a key intermediate metabolite of Krebs cycle [6]. 

PC deficiency is caused by mutations in the PC gene and depending on the residual activity of PC enzyme, three types have been described with variation in severity of clinical, biochemical manifestations, and prognosis [4,7]. Type A (infantile form) presents with mild to moderate lactic acidosis and most affected infants die in infancy or early childhood; Type B (severe neonatal form) presents in the neonatal period with severe features and most die in the first three months of life, and Type C (intermittent/benign form) presents with episodic metabolic acidosis during times of metabolic stress and affected neonates are developmentally normal or mildly delayed and have a normal life expectancy [1,4,8]. The neonate described in our case report had Type B, the severe neonatal form of PC deficiency.

Clinical presentation in PC deficiency is secondary to accumulation of toxic metabolites and limited replenishment of OAA to tricarboxylic acid (TCA) cycle and affecting the downstream metabolic pathways as described above. Infants with Type B PC deficiency presents in the early neonatal period with severe lactic acidosis, ketoacidosis, hypoglycemia, hyperammonemia, hepatic failure, and neurological manifestations like hypotonia, abnormal eye movements, seizures, pyramidal tract signs, and coma, and infants usually die within first couple months after birth [8]. Biochemical abnormalities typical of PC deficiency are lactic acidosis with increased lactate-to-pyruvate ratio, decreased 3-hydroxybutyrate to acetoacetate ratio, elevated blood concentrations of citrulline, proline, lysine, and ammonia, and low concentrations of glutamine, which the patient in the discussion also manifested [1]. 

In a case series describing nine patients with type B neonatal form of PC deficiency, Angels et al. noted the most common MRI brain finding to be white matter periventricular and subcortical high intensity and cysts and they also noted only four out of those nine patients described had hepatic manifestations. The infant in our report did not have any hepatic manifestations, and the brain MRI had loculated, septate large cysts along the caudothalamic notch and lateral aspect of the frontal horn bilaterally and diffuse white matter edema [9]. MR spectroscopy of the brain will show high levels of lactate and choline and low levels of N-Acetyl aspartate [1] which is described in our case. 

Differential diagnoses to consider in infants presenting with severe lactic acidosis include biotinidase deficiency, pyruvate dehydrogenase complex deficiency, respiratory chain disorder, TCA disorders, carbonic Anhydrase VA Deficiency, and gluconeogenic defects [1]. The diagnosis can be confirmed by the identification of biallelic pathogenic variants in PC gene on molecular genetic testing or by demonstrating the PC enzyme deficiency in fibroblasts or lymphoblasts [1,8]. 

There is no proven therapy currently available to modify or improve neurological symptoms in infants with PC deficiency. Providing an alternate source of energy and means of metabolizing pyruvate is the primary objective in managing these infants. A low-fat and high-carbohydrate diet and protein diet are recommended. Acute decompensation is managed through intravenous glucose-containing fluids, maintaining hydration, and correction of metabolic acidosis. Supplementation with thiamine, biotin, citrate, aspartic acid, triheptanoin, and lipoic acid have been used with variable success in improving the biochemical abnormalities and thus in somatic symptoms but did not alter the course of neurological damage and manifestations [1,8]. Prenatal molecular genetic testing and counseling, when available, should be provided to the parents when both pathogenic variants are identified in an affected family member.

## Conclusions

PC deficiency is a rare autosomal recessive (AR) genetic disorder. Infants presenting with severe lactic acidosis in the early neonatal period should be evaluated for PC as part of a differential diagnosis. It is associated with characteristic biochemical, MRI, and MR spectroscopy findings, and the diagnosis is confirmed either with molecular genetic testing or determining PC enzyme levels. There is no proven therapy so far and it is being managed symptomatically. Genetic counseling should be offered to parents when available.
